# New Knowledge in Analytical, Technological, and Biological Aspects of the Maillard Reaction

**DOI:** 10.3390/foods6060040

**Published:** 2017-05-26

**Authors:** Cristina Delgado-Andrade

**Affiliations:** Department of Physiology and Biochemistry of Animal Nutrition (EEZ-CSIC), Camino del Jueves, 18100 Granada, Spain; cristina.delgado@eez.csic.es; Tel.: +34-958-572757; Fax: +34-958-572753

The Maillard reaction (MR) is the non-enzymatic browning reaction that can occur both in foods and in living beings. Maillard reaction products (MRPs), some of them also known as advanced glycation end-products (AGEs), are a heterogeneous and complex group of compounds with many different in vitro and in vivo activities. From a technological point of view, the food industry has long been interested in this reaction, since its products can add desirable color, odor and taste to foods [1]. On the other hand, from a physio-pathological perspective, the in vivo effects of AGEs have attracted the attention of many researchers in the last years. Different reasons have motivated this interest. On the one hand, the human contact with MRPs may start in very early childhood, since the substitution of natural suckling of toddlers by the use of infant formulas has become increasingly common, and such formulas are usually rich in MRPs [2]. Moreover, the increase in life expectancy rates has been accompanied by the appearance of novel age-related pathologies, in which AGEs seem to be highly implicated [3,4]. Parallel to this, several socioeconomic factors have contributed as well. Young generations are less aware of traditional basic cooking techniques, or take no time to cook. A wide variety of precooked and highly processed foods are now sold, and these products have become a common part of many people’s diet. Another important factor is that of new dietary preferences, especially among the adolescent population. During adolescence, teenagers establish many of the dietary habits that will prevail for the rest of their lives, and start to choose foods for themselves, with fast food and snacks often prominent in this choice [5]. A study reported a Nε-(carboxymethyl)lysine (CML) intake of 11.28 mg/day by a group of adolescent males aged 11–14 years consuming a diet designed according to their food preferences, while the amount ingested when the diet was prepared with culinary treatments avoiding MR development was 5.36 mg/day (*p* < 0.05) [6]. In the same trial, the intakes of Amadori compounds and hydroxymethylfurfural (HMF) in both diets was tested. HMF consumption was 1.78 vs. 0.35 mg/day for the high and low MRP diets, respectively (*p* < 0.05), while the intake of Amadori compounds was 83.87 vs. 73.99 mg/day (*p* < 0.05) [7]. This dietary changes are happening to some extent not only in the adolescent population but also throughout the population, motivated in part by the fact that these foods often constitute an inexpensive option in the current economic situation [8]. In summary, the abovementioned factors have led to a substantial modification in the population’s dietary patterns. New food habits are introducing important amounts of MRPs and AGEs into the diet, mainly derived from bakery products and coffee. 

Aimed to detect the presence of these compounds in food matrices or in living beings, different analytical approaches have been designed, mostly based on chromatographic determinations. Although less reliable, some immunoassays have been also applied. The more analytical techniques advance, the higher the amount of new MRPs, AGEs, or promoters detected. Among those promoters, dicarbonyl compounds, generated by the MR as well as by caramelization and lipid oxidation, must be mentioned [9]. Their role as MR promoters lies in their ability to interact directly with amino residues or even with other intermediary compounds, leading to the formation of AGEs [10]. Dicarbonyl compounds are also responsible for the glycation of several biomolecules in vivo [11], and for promoting the formation of circulating AGEs.

Vlassara and co-workers were the first scientists who pointed that the total load of AGEs of the human body could derive from both in vivo synthesis and dietary exposure [12]. Uribarri et al. [13] studied a population of 90 healthy subjects and stated a positive and significant correlation between the level of serum CML and AGEs intake (measured by an immunological method). They concluded that dietary AGEs, abundantly present in the Western diet, contribute significantly to the body’s pool of AGEs. For this reason, glycation products have been classified as endogenous and exogenous AGEs, and their final balance in the organism is a key factor in the development and progress in different degenerative and chronic diseases [4]. Maintaining a balanced and varied diet, not only in the food consumed but also in the way it is processed, appears to be the best strategy to control the negative effects and to preserve the positive actions of dietary MRPs [4]. An interesting review within this special issue revises the impact of the diet on the accumulation of AGEs in the human body [14]. Among its conclusions, the importance of dietary adjustment is highlighted as preventive therapy to control AGEs accumulation in the organism. A “slow carb” diet can help to decrease glucose uptake and thus control its blood level. On the other hand, since the carbonyl stress involved in the formation of endogenous AGEs is closely linked to oxidative stress, any diet that will improve the oxidative status will potentially have the additional benefit of reducing the formation of endogenous AGEs. An additional strategy to slow down glycation is to trap in vivo dicarbonyl compounds using natural compounds extracted and purified from foods which have chemical affinity by dicarbonyl compounds or an indirect ability to promote the mechanism of detoxification for these compounds [15]. The study of all these compounds and their mechanism of actions, as well as the design of new strategies to prevent in vivo effects of AGEs accumulation, will be studied in the future and will undoubtedly be a key factor to improve the quality of life for the elderly.
